# Implementation of Human-Machine Synchronization Control for Active Rehabilitation Using an Inertia Sensor

**DOI:** 10.3390/s121216046

**Published:** 2012-11-22

**Authors:** Zhibin Song, Shuxiang Guo, Nan Xiao, Baofeng Gao, Liwei Shi

**Affiliations:** 1 Department of Intelligent Mechanical Systems Engineering, Kagawa University, Hayashi-cho, Takamatsu 761-0369, Japan; E-Mails: guo@eng.kagawa-u.ac.jp (S.G.); xiao@eng.kagawa-u.ac.jp (N.X.); gaobaofeng@eng.kagawa-u.ac.jp (B.G.); shi@eng.kagawa-u.ac.jp (L.S.); 2 Automation College, Harbin Engineering University, 145 Nantong Street, Harbin, Heilongjiang 150001, China

**Keywords:** exoskeleton device, motion tracking, backdrivability, double close-loop control

## Abstract

According to neuro-rehabilitation practice, active training is effective for mild stroke patients, which means these patients are able to recovery effective when they perform the training to overcome certain resistance by themselves. Therefore, for rehabilitation devices without backdrivability, implementation of human-machine synchronization is important and a precondition to perform active training. In this paper, a method to implement this precondition is proposed and applied in a user’s performance of elbow flexions and extensions when he wore an upper limb exoskeleton rehabilitation device (ULERD), which is portable, wearable and non-backdrivable. In this method, an inertia sensor is adapted to detect the motion of the user’s forearm. In order to get a smooth value of the velocity of the user’s forearm, an adaptive weighted average filtering is applied. On the other hand, to obtain accurate tracking performance, a double close-loop control is proposed to realize real-time and stable tracking. Experiments have been conducted to prove that these methods are effective and feasible for active rehabilitation.

## 1. Introduction

Robot mediated rehabilitation is being studied by many researchers with the development of robotics, mechatronics and neuroscience [1]. In the view of neuroscience, passive training and active rehabilitation training influence the plasticity and recovery of the brain following a stroke [2,3]. Moreover, in active training, progressive resistance was proved beneficial in improving muscular strength in the elderly [4] and chronic myopathies [5]. In rehabilitation robots for the upper limbs, there are two main strategies: one is the end-effector type and the other is the exoskeleton type. One of the earliest robotic rehabilitation systems of the end-effector type, called MIT-MANUS, was developed by Krebs *et al*. [6,7]. It allows two degrees of freedom (DoFs) for upper limb movement, including wrist, elbow and shoulder movements, by performing task-oriented training. It can provide passive and active rehabilitation to stroke patients and it has improved motor function in the hemiparetic upper limbs of acute and chronic stroke patients in clinical trials [8]. In 1997, with the cooperation of Stanford University and the Rehabilitation Research and Development Centre, another rehabilitation system named Mirror-image motion enabler (MIME) was developed [9,10]. This robot can work in pre-programmed position and orientation trajectories. It can also provide mirror movement that affected upper limbs can perform like the movement of the intact upper limb. Different from it, Guo and Song at Kagawa University have developed a coordination rehabilitation system for bilateral upper limbs using a haptic device and an inertia sensor [11]. Gentle/s was a three-year project funded by the European Commission to develop machine-mediated therapies for neurorehabilitation of people with strokes. Gentle/s had the aim to improve the quality of treatment and reduce costs [12]. These robots are a typical paradigm of end-effector type rehabilitation robots and have successfully provided patients enough assistance and training range. However, they cannot perform individual joint training, so that arm posture cannot be confirmed and there is even a risk of joint injury to the stroke patients [13]. The exoskeleton robots that have recently been developed quickly for upper and lower limb rehabilitation solve this problem. One typical exoskeleton device, MEDARM, developed by the Canadian Institutes of Health Research (CIHR), is based on a cable driven curved track mechanism that provides independent control of all five major DoFs in the shoulder complex [14]. The ARMin [15] is an exoskeleton device with six independently actuated DoFs and one coupled DoF. It can provide passive and active rehabilitation to stroke patients and significantly improve motor function of the paretic arm in some stroke patients, even those in a chronic state [16]. These systems have advantages in upper limb rehabilitation, including providing multiple rehabilitation strategies and enough range of movement, but they still have some disadvantages. For example they are heavy and not suitable for home-rehabilitation. We have designed an upper limb rehabilitation system with a therapist’s guidance, which is compact and portable and has the potential to be used for home rehabilitation.

In previous work, we have realized passive training by using this device. It is available for stroke patients who have lost motor function in the upper limbs [17], but for active training, it is not easy to implement it because of the intrinsically mechanical structure of the exoskeleton device. Firstly, the wearability and portability require that the device, including the actuators, be light enough. Secondly, in passive training, the device should exert enough torque to assist patients in performing the training, therefore the high ratio gearheads have to be used. Meanwhile, it induces high friction and inertia so the device is intrinsically non-backdrivable. Last, it is a precondition to perform active training that the device be backdrivable. The current method to improve the backdrivability of a device is the use of admittance control, which can drive the device according to the contact force between the human and the device [18,19]. However, it is also very difficult to obtain the accurate contact force for our device, because the contact condition between the ULERD and human limbs is very complicated. Therefore, in this paper, we propose a method to achieve the precondition of active training via driving the device to track the motion of human limb. We use an inertia sensor (MTx) [20] to track the motion of the upper limb to make the device backdrivable to implement human-machine synchronization under the condition that the connection between human limb and the device is flexible and not rigid. This proposed method can also be used in other human machine interactions when the device is intrinsically non-backdrivable.

This paper is organized as follows: it first introduces the background and related research. In Section 2, the proposed rehabilitation system is presented in detail. The proposed control methodology to implement tracking performance in real time is shown in Section 3. Experiments and results are presented in Section 4. The last section presents the discussion and conclusions of this paper.

## 2. Overview of the Proposed System

### 2.1. Mechanism of Exoskeleton Device Designed

**Figure 1.**
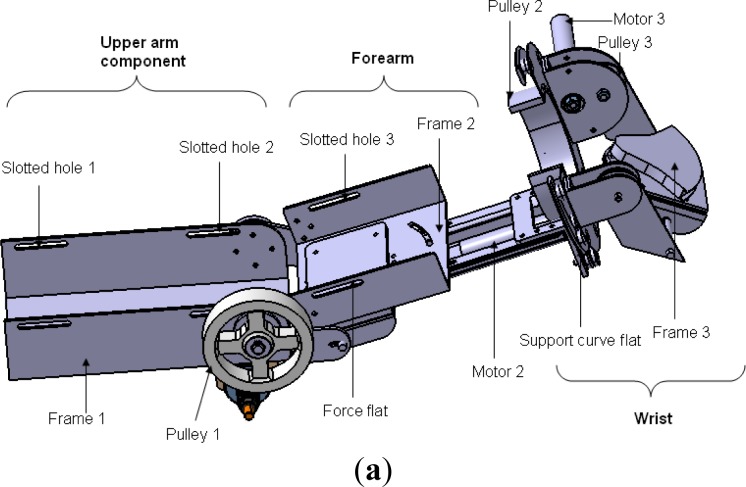

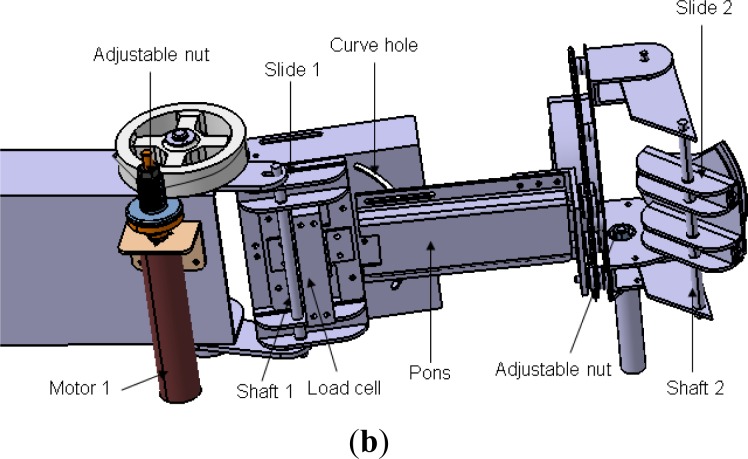
(**a**) The upper view of the ULERD (**b**) The lower view of the ULERD.

The motivation of the ULERD design is to provide multiple rehabilitation strategies to patients with motor dysfunction to recover the motor functions of the upper limbs, including elbow and wrist joints. The basic design structure of the ULERD from an upper view is depicted in Figure 1. Three active DoFs were designed in the elbow and wrist, including the elbow flexion/extension, forearm pronation/supination and wrist flexion/extension [21]. These three DoFs are both actuated and sensorised. On the other hand, four passive DoFs were added, including two DoFs (one is rotation and the other is translation) in the elbow joint, another two in the wrist joint considering some factors, for example, variation of flexion/extension axis (FEA) [22], personalized otherness in physical dimension of the joint and correlating the misalignment between the device and human limb. Two passive rotational DoFs are sensorised with potentiometers and they can be locked or unlocked according to different cases.

The ULERD is comprised of three parts: upper arm, forearm and wrist. In three actuated DoFs, power derived from motors is transmitted to a drive pulley through stainless steel wire ropes after the rotational velocity is decreased via high ratio gearheads. This kind of transmission structure can not only provide enough torque for passive training, but also decrease the backlash generated by gearheads which is undesirable in active training [23]. The motor in the elbow joint was mounted perpendicularly to the axis of the upper arm considering the stability. The upper limb is fixed to the device using several elastic belts passing through the slotted holes on the upper arm part and forearm part. The palm can also be fixed on the wrist part using the elastic belt. The wearing design of the ULERD aims to make users wear it conveniently by themselves. The distance between the elbow joint of the ULERD and wrist part can be moderately adjusted in accordance with different users, and the angle between the upper arm part and forearm part can also adjusted.

### 2.2. Actuator in Elbow Joint of the ULERD

It is important to choose the actuator in a portable and wearable exoskeleton device for upper limb rehabilitation. For such a device, high power-to-weight ratio and high bandwidth are desirable actuator qualities. Electrical actuators have a lower power-to-weight ratio than pneumatic actuators, but offer very high bandwidth [24]. High bandwidth is a crucial factor for active training, so we chose a Maxon BLDC motor because of its lighter weight, more compact size, higher power density and torque density than conventional motors (Table 1).

In passive rehabilitation mode, the device should be able to exert enough torque; meanwhile, the training is performed slowly, so a gearhead with high ratio can be used. The safety requirements are also very important for a rehabilitation device, so a mechanical mechanism was designed to prevent overload during rehabilitation. The helical capstan shaft is set apart from the motor shaft during overload. In detail, the axle sleeve of the motor is connected to the helical capstan shaft by the friction derived from adjusting an outer thumbnut (Figure 1(b)).

### 2.3. The Inertia Sensor

**Figure 2.**
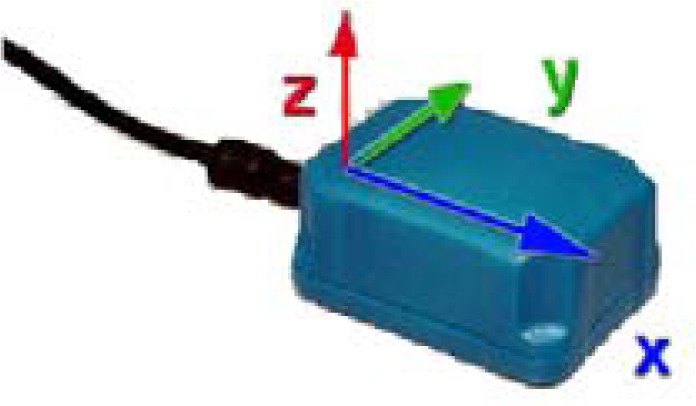
The inertia sensor (MTx).

The adopted inertia sensor (MTx sensor, Figure 2) is developed by Xsens [20]. Its unit combines a tri-axial accelerometer, a tri-axial magnetometer and a tri-axial gyroscope. With a sensor fusion algorithm, the sensor is able to distribute the raw sensor data and a drift free orientation. It can be used in detection of “pitch”, “yaw” and “roll” directly after calibration.

## 3. Control Methodology

**Figure 3.**
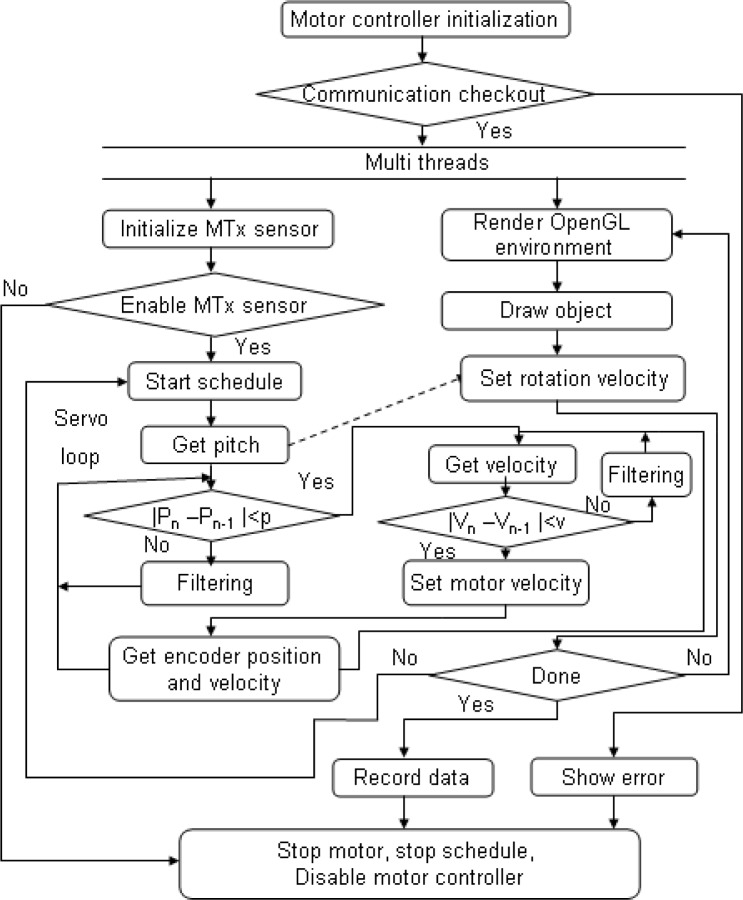
The flow chart of the program.

When speaking of neurorehabilitation, visual stimulation and visual feedback are important, therefore, a user interface was created based on OpenGL, which can monitor the status of a user’s limb, including rotational angles and velocity. In order to monitor the motion and the device’s tracking performance in real time, a multi-thread was utilized in the program, in which reading and setting angle or velocity are of the higher priority than rending the OpenGL graph (Figure 3).

In this paper, the typical tracking performance was implemented on elbow flexion and extension in the sagittal plane, so the motion of forearm can be detected by measurement of the pitch angle of the inertia sensor. The real-time motion tracking system is implemented in the Visual Studio 2005 environment, and the computer used is a HP workstation equipped with a Pentium 4 (3.4 GHz) CPU.

### 3.1. Adaptive Weighted Filtering

The values derived from sampling of angle and rotation velocity by the MTx sensor are not ideal because they contain some noise, particularly for the rotation velocity. Therefore, it is necessary to process these raw values. The rotational angle or rotational velocity of the user’s limb should be smooth because of the mechanism of a human’s limb, so some predefined values derived from many repeated experiments are set as a threshold value to detect the validity of data. If a value derived from sampling is over the threshold value, it will be modified through filtering, but it is not sufficient to make sure all the data derived are valid. There are some kinds of filtering that can be realized in the program [25–27], but few of them can satisfy the requirement of real-time control. Some splendid filtering methods require lots of calculation. In this paper, we used a simple adaptive weighted averaging filtering which is calculated with only four values each one time (Equation (1)):

(1) $$\bar{y}=1/N\sum_{n=1}^{N}A\cdot y_{n}$$

where *y_n_* is the raw signals derived from sampling; *N =* 4 in this paper; *A* is the weighted coefficient which is calculated according to *y_n_*.

**Figure 4.**
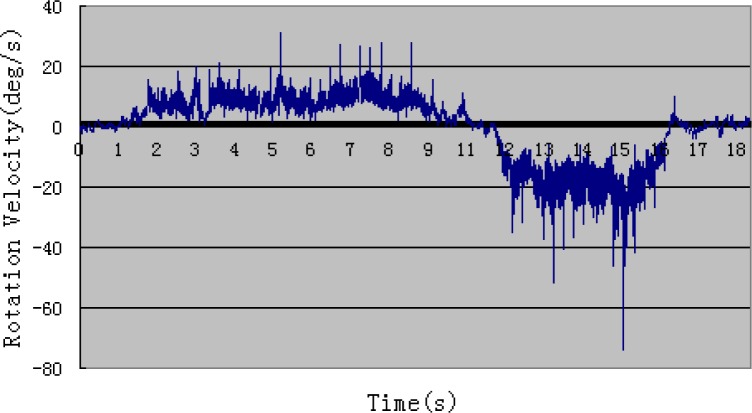
Rotational velocity before filtering.

**Figure 5.**
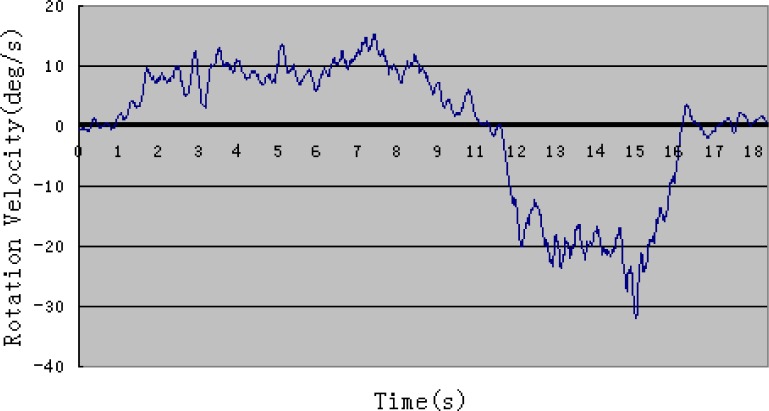
Rotation velocity after filtering.

Figures 4 and 5 show one example of rotation velocity derived before filtering and after filtering. It is obvious that the value after filtering become smoother.

### 3.2. Double Closed-Loop Control

**Figure 6.**
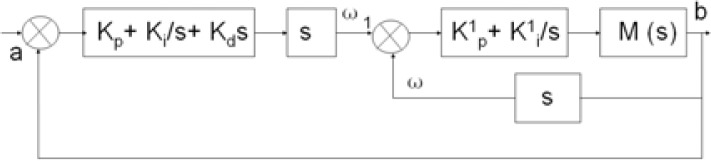
Double closed-loop control block chart.

In order to obtain precise motion tracking, a double closed loop control is adapted on the basis of the previous research. The control block diagram is shown in Figure 6.

The inner loop is a velocity closed loop which is implemented in the motor driver. The outer loop is a closed-loop of rotational angle where the rotational angle of a user’s limb is the target and the rotation angle of the device is the value under control. *a* is the pitch angle of the inertia sensor namely the rotational angle of user’s forearm. *b* is the angle of the device detected using the encoder. *ω*_1_ is pitch angle velocity of user’s forearm. *ω* is output velocity of motor. *kp*, *k_i_*, and *k_d_* are the coefficients of PID control of outer loop and the values are set as 238, 1,440, 12, respectively, which can be obtained from repeated experiments. *k^1^_p_* and *k^1^_i_* are the coefficients of PI control of inner loop and the values are set as 242 and 39, respectively. The system transfer function can be calculated with Equation (2). Though the current loop is not mentioned in this paper, the current is also detected to make sure that torque exerted by motor is at a safe level:

(2) $$G(s)=\frac{K_{1}K_{2}}{1+K_{1}+K_{1}K_{2}}$$

where:

$$\begin{array}{c} K_{1}=s(K^{1}{}_{p}+K^{1}{}_{i}/s)M(s)\\ K_{2}=K_{p}+K_{i}/s+k_{d}s \end{array}$$

## 4. Experiments and Results

### 4.1. Motion Detection and Coefficients Calculation of the System Based on the Disconnected Experiment

**Figure 7.**
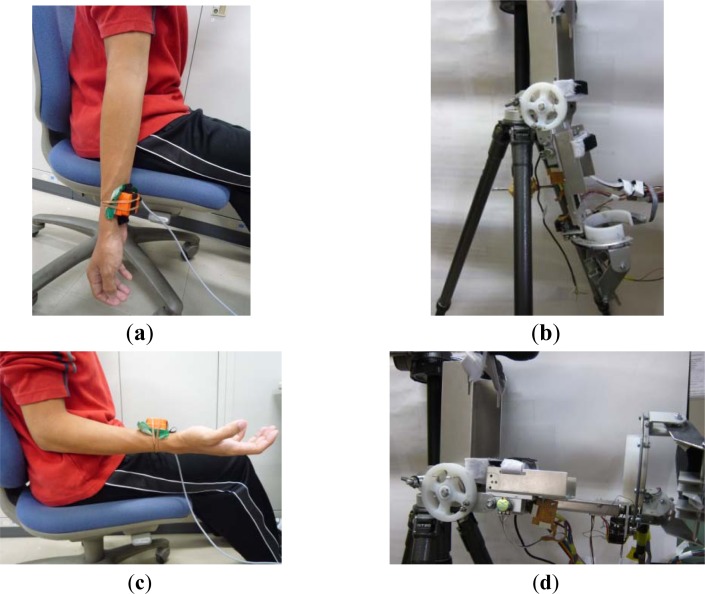
The disconnected experiment. (**a**) The subject’s forearm on the vertical plane with the inertia sensor on his wrist; (**b**) The ULERD is extended to the vertical plane; (**c**) The subject’s forearm on the horizontal plane with the inertia sensor on his wrist; (**d**) The ULERD is extended to the horizontal plane.

Before performing a real-time motion tracking experiment, the coefficients of the system methodology can be obtained by calibrating the inertia sensor and the ULERD in disconnected experiments. In this phase, the inertia sensor is fixed onto a healthy subject’s upper arm near to his wrist. The subject is required to perform elbow extension/flexion at a slower speed with the upper arm fixed to estimate the training for stroke patients (Figure 7(a,c)). The ULERD is fixed on a tripod with component of upper arm fixed (Figure 7(b,d)). The aim of experiment is to realize the synchronized motion of the ULERD with the subject’s forearm during elbow flexion and extension. The optimized control coefficients can be calculated by input and output of system after many experiments according to Equation (2).

Generally speaking, the range of movement of human elbow is from 0° (Figure 7(a)) to 135° during flexion and extension. In order to accomodate more patients, in this experiment the subject was required to perform the motion from 0° to 100°. For safety, if the device rotates over this range, the motor will be stopped at once. On the other hand, if the device rotates above the safe velocity, the motor will also stop at once, which can be set according to the therapist’s experience and patients’ physical condition.

**Figure 8.**
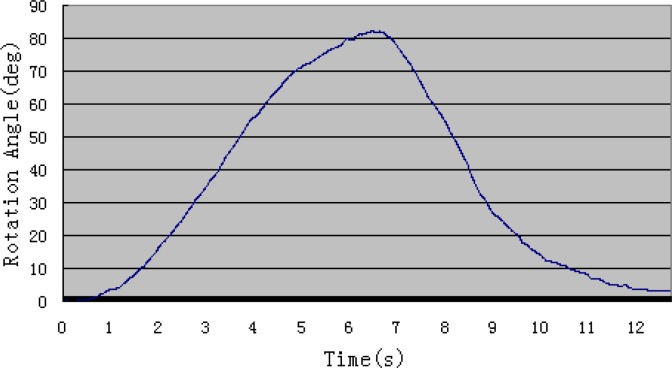
Rotational angle of the upper arm with the inertia sensor.

**Figure 9.**
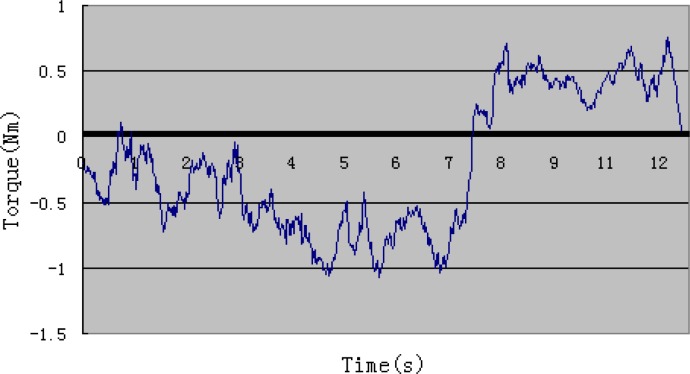
Motor current.

Figure 8 shows the angle value derived from the inertia sensor when the subject rotates his forearm around the elbow joint without wearing the ULERD in the sagittal plane. In this system, the force sensor is not adopted, and we have considered an alternative to estimate the resistance during experiments and it will also be used to ensure safe performance. That is the motor current, because of the relationship between the torque and motor current. Therefore, the filtered current *I_m_* is used to assess the resistance of the elbow joint. The torque exerted on the motor can be obtained in terms of current (Equation (3)):

(3) $$T={\mathrm{nlI}}_{m}$$

where *T* is the torque exerted on motor. *l* is the coefficient of motor (here, *l* = 23.5 mNm/A), *n* is the ratio of the gearhead. Figure 9 shows one example of filtered torque when the elbow joint of the exoskeleton device rotates.

### 4.2. Real Time Motion Tracking of Upper Limbs Based on Connected Experiments

**Figure 10.**
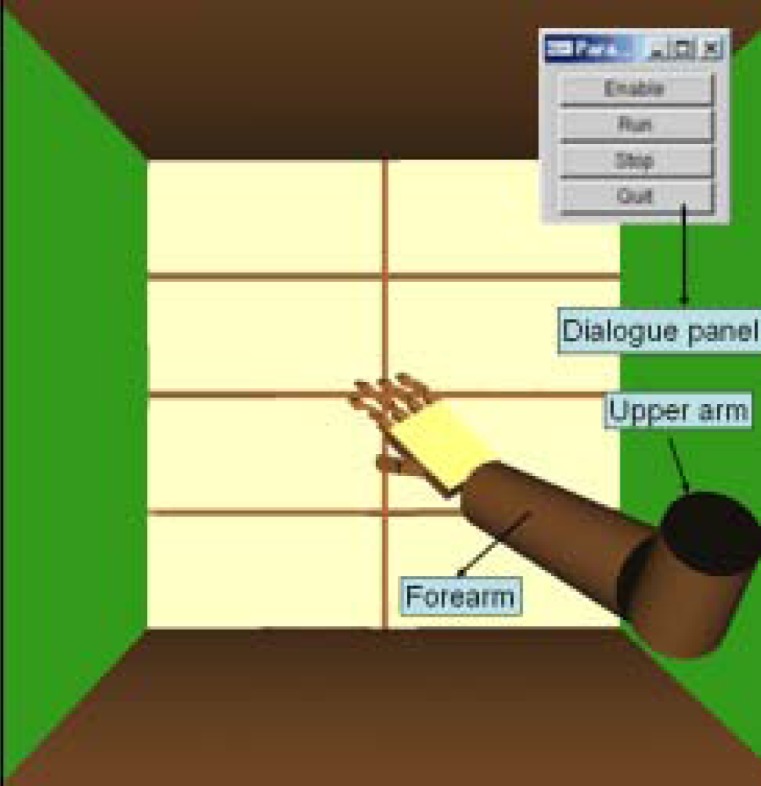
Experimental virtual environment.

A visual user interface based on OpenGL is created, in which two linkages stand for upper arm and forearm. There are also some manipulation buttons on the monitor, including “enable”, “run”, “stop” and “quit”, which are convenient for the user to control the situation of the task (Figure 10).

**Figure 11.**
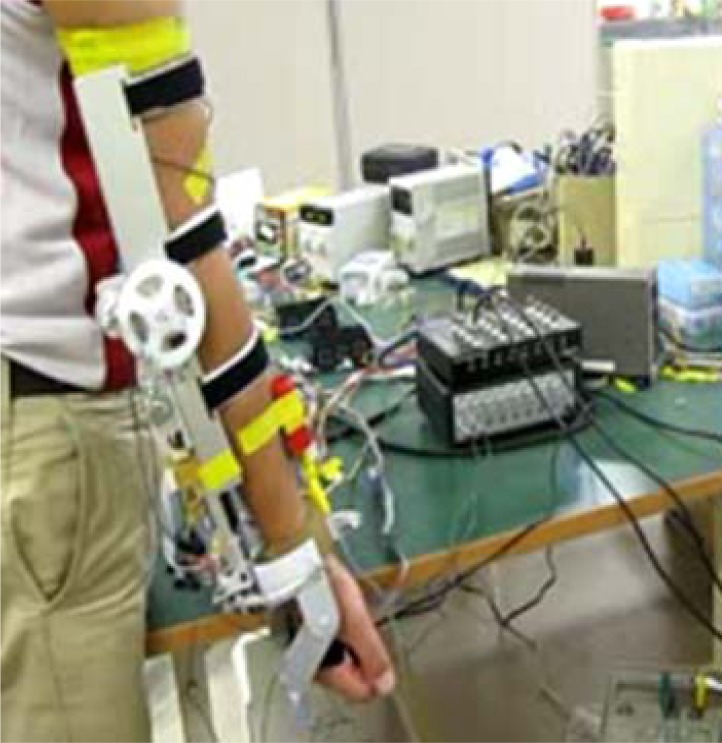
Subject wears the exoskeleton device with the MTx sensor fixed on his upper arm.

After confirming the control coefficients, the tracking of upper limb movement will be performed in real-time based on the connected experiments. The subject sits on a chair facing the monitor of a computer. The ULERD is fixed onto his right arm with some elastic belts. The inertia sensor is mounted on the upper arm near the wrist joint (Figure 11). The purpose of this experiment is to realize the synchronized motion between upper arm and the device so that the subject can hardly feel the resistance while performing the experiment.

As long as it can be realized, a reasonable resistance could be generated and provided to patients by setting a certain delay in the tracking motion and the active rehabilitation can thus be implemented. In the experiments, the subject is also required to perform elbow flexions and extensions. During this process, the subject keeps looking at the monitor.

**Figure 12.**
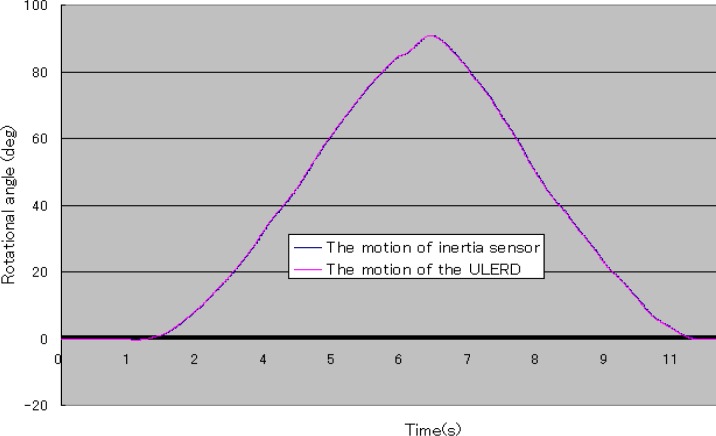
Rotational angle of the inertia sensor and the ULERD.

In Figure 12, the blue curve stands for the rotational angle of the subject’s forearm detected by the inertia sensor and the pink curve stands for the rotational angle of the ULERD. Both trajectories are almost superposed, which indicates that the proposed method is effective and the tracking performance is implemented well in real time. Comparing Figure 12 with Figure 8, we can know that the subject’s performance of the rotational forearm motion while wearing the exoskeleton device and the inertia sensor is almost the same as the case of not wearing the exoskeleton device. This indicates that the ULERD can perform compliantly with the motion of subject’s upper limb without constraints. Therefore, this prototype of exoskeleton device has the potential to be used in active rehabilitation.

**Figure 13.**
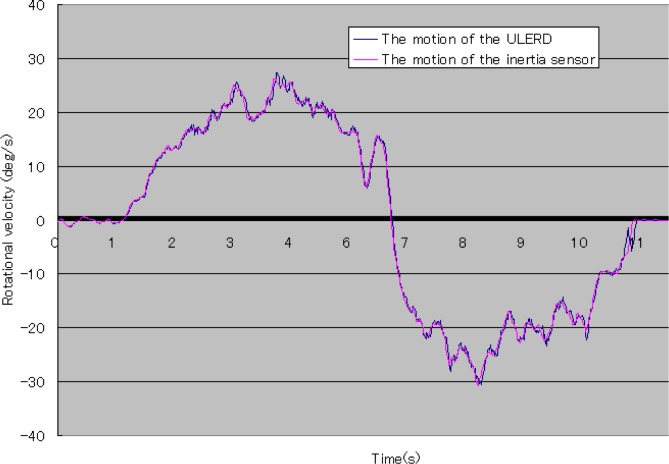
Rotational velocity of the inertia sensor.

In Figure 13, the blue curve stands for the rotational velocity of the ULERD and the pink curve stands for the rotational velocity of the inertia sensor. We can see both trajectories are also the same. This means that the velocity of motion of the user’s forearm is the same as that of the device and the tracking performance can be implemented in real time. It also indicates that the proposed method can implement the human-machine synchronization using the ULERD and the inertia sensor.

**Figure 14.**
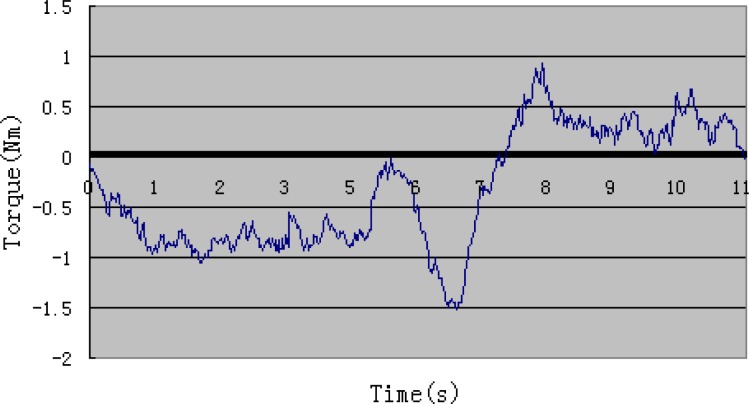
Output torque of motor.

Figure 14 shows the output torque of the motor during the experiment. According to Figure 9, we can learn that the output torque becomes a little higher around the sixth second, which is because the elastic belts prevent skin’s stretching when the forearm is perpendicular to the upper arm. Resistance of the structure influences subject’s performance only in a small range. The surveillance of output torque is important for safety reasons. It is also a reference in active training.

## 5. Discussion

Robot-mediated rehabilitation has been developing quickly in recent times, particularly exoskeleton devices. However, the current exoskeleton devices for upper limb rehabilitation are almost always heavy and large so that they are barely used in home-rehabilitation. In our research, we introduced a light and portable exoskeleton device for upper limb rehabilitation (ULERD). As a device for upper limb rehabilitation, two basic training strategies should be provided to patients: passive training and active training. In previous research, passive training using the ULERD has been discussed. The basic precondition of active rehabilitation is that a user can move his limb without resistance wearing the ULERD, namely human-machine synchronization. In this paper, we focused on this preliminary research for the active rehabilitation, which is implementation of tracking of the motion of the ULERD to the motion of user’s limb. On the other hand, because the ULERD is non-backdrivable, we used an inertia sensor to detect the motion of the user’s limb and then control the ULERD to move along with it based on a double control algorithm in real time. Meanwhile, to get optimized motion signals of the inertia sensor, an adaptive weighted average filtering was adapted. The aim of this preliminary research is to implement the non-constraint motion for the subject when wearing the ULERD. The feasibility condition to use this method is that the connection between the human limb and the device be flexible and not rigid. In future active training, desired resistance can be exerted on the user by adjusting the relative motion between the user’s limb and the ULERD.

## 6. Conclusions

In order to implement active training for a rehabilitation device without backdrivability, some basic research to implement the needed human-machine synchronization is presented in this paper, which focused on elbow flexion and extension performed by a healthy subject. The experiments conducted include a disconnected experiment and a connected experiment. The aim of the disconnected experiment is to obtain the optimized methodology coefficients and the connected experiment aims to evaluate the efficacy of the proposed method. According to the results of the connected experiment, the trajectory of rotational angle of the inertia sensor is almost the same as that of rotational angle of the ULERD. The subject can move his forearm with less resistance over a large range. Therefore, it verifies that this method can realize the human-machine synchronization and has the potential to be used in active rehabilitation. In the future, we will implement generation of some reasonable resistance values according to a predefined virtual model [28].

## Figures and Tables

**Table 1.** Characteristics of the actuator combination.

Weight	85 g
Voltage	24 V
Max. continuous torque	14.2 mNm
Max. continuous speed	50,000 rpm
Encoder precision	512
Gear mechanism	231:1
